# The Effect of Cholesterol on Membrane-Bound Islet Amyloid Polypeptide

**DOI:** 10.3389/fmolb.2021.657946

**Published:** 2021-04-22

**Authors:** Mikkel Christensen, Nils A. Berglund, Birgit Schiøtt

**Affiliations:** ^1^Department of Chemistry, Aarhus University, Aarhus, Denmark; ^2^Interdisciplinary Nanoscience Center (iNANO), Aarhus University, Aarhus, Denmark; ^3^Sino-Danish Center for Education and Research, Beijing, China

**Keywords:** cholesterol, amylin, simulations, diabetes, aggregation, amyloid

## Abstract

Islet amyloid polypeptide (IAPP) is a proposed cause of the decreased beta-cell mass in patients with type-II diabetes. The molecular composition of the cell-membrane is important for regulating IAPP cytotoxicity and aggregation. Cholesterol is present at high concentrations in the pancreatic beta-cells, and in-vitro experiments have indicated that it affects the amyloid formation of IAPP either by direct interactions or by changing the properties of the membrane. In this study we apply atomistic, unbiased molecular dynamics simulations at a microsecond timescale to investigate the effect of cholesterol on membrane bound IAPP. Simulations were performed with various combinations of cholesterol, phosphatidylcholine (PC) and phosphatidylserine (PS) lipids. In all simulations, the helical structure of monomer IAPP was stabilized by the membrane. We found that cholesterol decreased the insertion depth of IAPP compared to pure phospholipid membranes, while PS lipids counteract the effect of cholesterol. The aggregation propensity has previously been proposed to correlate with the insertion depth of IAPP, which we found to decrease with the increased ordering of the lipids induced by cholesterol. Cholesterol is depleted in the vicinity of IAPP, and thus our results suggest that the effect of cholesterol is indirect.

## Introduction

Islet amyloid polypeptide (IAPP, also known as amylin) is co-secreted with insulin (Lukinius et al., 1989) from the beta cells of the pancreatic islets, and together with insulin it regulates the glucose metabolism (Lutz, 2010, 2012; Hay et al., 2015). In most patients with type-II diabetes mellitus (T2DM) IAPP aggregates to form islet amyloid (Maloy et al., 1981; Westermark, 1995; Westermark et al., 2011). IAPP aggregation is cytotoxic and contributes to the loss of beta cell associated with progressed T2DM (Abedini and Schmidt, 2013; Cao et al., 2013). While T2DM is initially characterized by a reduced insulin response, the loss of beta cells decreases the production of insulin and IAPP and thus further reduces the regulation of the glucose metabolism (Höppener et al., 2000). The cytotoxic mechanism of IAPP toward beta cells is unclear, but it has been hypothesized to be related to both membrane damage, inflammation, receptor interactions, oxidative stress, mitochondrial dysfunction, and inducing defects in autophagy (Abedini and Schmidt, 2013; Milardi et al., 2021).

**FIGURE 1:**
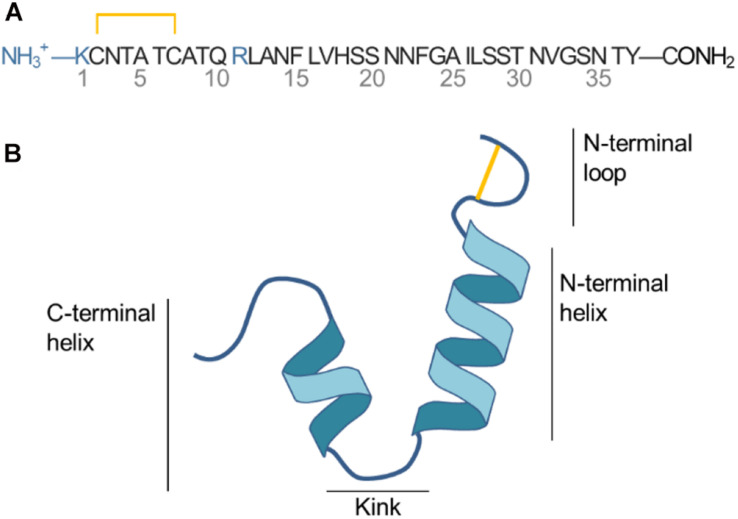
**(A)** Sequence of IAPP. **(B)** Sketch of the secondary structure of IAPP in the helical kinked conformation.

Islet amyloid polypeptide is a 37 residue peptide with a net positive charge of +3 (N-terminal, Lys1 and Arg11) at physiological pH, as illustrated in Figure 1A. A histidine residue at position 18 can be protonated to carry a positive charge depending on the pH (Abedini and Raleigh, 2005). A disulfide bridge connects Cys2 and Cys7 and loops the N-terminus. IAPP is an intrinsically disordered peptide, meaning that it varies between multiple folded and unfolded conformations in solution as revealed by NMR experiment and CD spectroscopy (Williamson and Miranker, 2007; Williamson et al., 2009). At hydrophobic/hydrophilic interfaces (e.g., the interface of a phospholipid bilayer or a micelle) IAPP can stabilize in a conformation with amphiphilic α-helices (Knight et al., 2006). Based on structural ensembles solved from NMR experiments of micelle bound IAPP the α-helical structure of IAPP can be either straight (helical from residue 5 to 28) (Patil et al., 2009) or kinked (with a turn at residue Ser19 to Phe23, as sketched in Figure 1B) (Nanga et al., 2011). The helicity of membrane bound IAPP has also been observed on large unilamellar vesicles (LUVs) using circular dichroism (Jayasinghe and Langen, 2005; Knight et al., 2006; Engel, 2009; Khemtémourian et al., 2011; Lee et al., 2012).

Few structures of the mature fibrils are available; Wiltzius et al. (2008) has proposed a structure based on X-ray crystallography of two IAPP segments and Luca et al. (2007) has proposed a structure using constraints from solid-state nuclear magnetic resonance spectroscopy. Common for the fibril structures is the stacking of IAPP peptides along the fibril axis, with beta sheets forming between adjacent peptides. The individual peptides have a turn spanning from residue His18 to Phe23 (Luca et al., 2007).

Membrane damage induced by IAPP oligomers or amyloid is a proposed mechanism of cytotoxicity (Raleigh et al., 2017). Several studies have investigated membrane induced IAPP aggregation, since islet amyloid is located at the beta cell surfaces. The cell membrane is complex and contains many different lipids (Nicolson, 2014) and the aggregation is very dependent on the lipid composition, as indicated from observed lipid dependent changes in fibril morphology and the rate of formation (Sciacca et al., 2018). Dye-leakage and Thioflavin-T (ThT) assay fluorescence experiments are commonly used to study IAPP aggregation on model membranes to investigate the aggregation rate and membrane perforation of IAPP (Zhang et al., 2017). The aggregation rate and membrane perforation of IAPP is drastically increased in model systems with zwitterionic phosphatidylcholine (PC) lipids and anionic lipids such as phosphatidylglycerol (PG) and phosphatidylserine (PS) (Sciacca et al., 2008; Zhang et al., 2017). Working toward more realistic membrane models, several studies have recently focused on the interactions between IAPP and membrane cholesterol (CHOL) (Li et al., 2016; Zhang et al., 2017, 2018; Hao et al., 2018). CHOL is highly abundant in mammalian plasma membranes and it has been shown to be important for the function of many membrane associated peptides and proteins (Grouleff et al., 2015), transporting both ions and molecules e.g., the sarco/endoplasmic reticulum Ca^2+^-ATPase (SERCA) (Li et al., 2004), the dopamine transporter (Zeppelin et al., 2018), aquaporins (Jacob et al., 1999), and peptides such as amyloid-β (Ji et al., 2002; Habchi et al., 2018). CHOL changes the fluidity and order of lipid membranes thereby indirectly affecting proteins that are embedded or attached to the membrane, or directly by interacting with the protein or peptide in a manner that affects structure and function (Grouleff et al., 2015; Zeppelin et al., 2018). Some experiments points toward an inhibiting role of CHOL toward IAPP aggregation, whereas other studies indicate that CHOL accelerate IAPP aggregation and cytotoxicity (Li et al., 2016; Zhang et al., 2017; Hao et al., 2018).

Zhang et al. (2017) studied the membrane interactions of IAPP in large unilamellar vesicles, probing amyloid formation using ThT fluorescence and membrane permeabilization from dye leakage experiments. They found that CHOL slows down amyloid formation and dye leakage in membranes consisting of 1,2-dioleoyl-*sn*-glycero-3-phosphatidylcholine (DOPC or PC), 10% 1,2-dioleoyl-*sn*-glycero-3-phosphatidylserine (DOPS or PS), and CHOL concentrations of 20 and 40%. They also showed that high concentrations of anionic DOPS lipids (above 25%) diminish the effect of CHOL (Zhang et al., 2017). In a follow-up study, Zhang et al. further investigated the effect of changing the type of sterol. They investigated the membrane binding, perforation, and amyloid formation on membranes with eight different types of sterols (Zhang et al., 2018). They noticed that for the fraction of vesicles that bound IAPP, the membrane leakage, and amyloid formation was inversely correlated with the ordering of the lipids in the vesicles. This points to the conclusion that the effect of CHOL is indirect, thus dictated by changing membrane properties, rather than by a direct interaction of CHOL with IAPP. In a study by Yang et al. the interaction between the 19 N-terminal residues of IAPP and small unilamellar vesicles (SUVs) consisting of DPPC and CHOL was studied. Using ^31^P-NMR they estimated the dissociation constant of IAPP from the vesicle for different concentrations of CHOL, and found that the N-terminal fragment bound stronger to membranes with increasing concentrations of CHOL. Based on mutational studies they hypothesized a direct interaction between IAPP and CHOL by CH−π interactions between Phe15 and CHOL (Li et al., 2016). Sciacca et al. (2016) studied the effect of CHOL in LUVs with DOPC, DPPC, and CHOL in a ratio known to form lipid nanodomains with the formation of liquid ordered domains with saturated lipids and CHOL and liquid disordered domains containing unsaturated lipids. A DOPC:DPPC ratio of 1:2, CHOL accelerated amyloid formation and leakage with a CHOL content of 20 or 40%, compared to pure DOPC vesicles, as shown from ThT fluorescence and dye leakage experiments, respectively. Based on atomic force microscopy imaging they found that the disruption of the membrane occur at the boundary between the l_*o*_ and l_*d*_ domains (Sciacca et al., 2016). Several studies point to the same conclusion: Membranes with CHOL-induced domain formation accelerate IAPP aggregation and membrane damage, and IAPP will have a high affinity for the boundary between the l_*o*_ and l_*d*_ domain (Trikha and Jeremic, 2011; Caillon et al., 2014). Conversely, in membranes without domain formation, CHOL inhibits the aggregation and membrane damage induced by IAPP (Zhang et al., 2017).

Interactions between IAPP and phospholipid membranes have previously been investigated using MD simulations. Many of these studies have focused on simple membrane compositions composed of zwitterionic phospholipids and anionic lipids such as PG or PS (Dong et al., 2018). Simulation studies have previously been performed with CHOL, however, these studies have been coarse-grained, and thus not able to investigate the details of the interactions between IAPP and CHOL. In this study we apply atomistic molecular dynamics (MD) simulations to investigate the interaction between IAPP and lipid bilayers with various lipid compositions. With MD simulations, we achieve atomistic details about the interactions between IAPP and lipid bilayers with a mixture of CHOL, DOPC, and DOPS lipids. Further analysis of the effect of these lipids on membrane-bound IAPP are performed. Lipid rafts and the effect of these on membrane-bound IAPP will not be discussed further in this paper, since the focus herein is on the effect of CHOL on IAPP in membranes that do not form lipid rafts. Due to the limited timescale of MD simulations, the investigations are limited to the membrane-bound helical state of a monomer, and not the effect of the lipids on the oligomerization and resulting conformation transitions, which was recently described from simulations in membranes without CHOL (Skeby et al., 2016; Christensen et al., 2017).

## Materials and Methods

**FIGURE 2:**
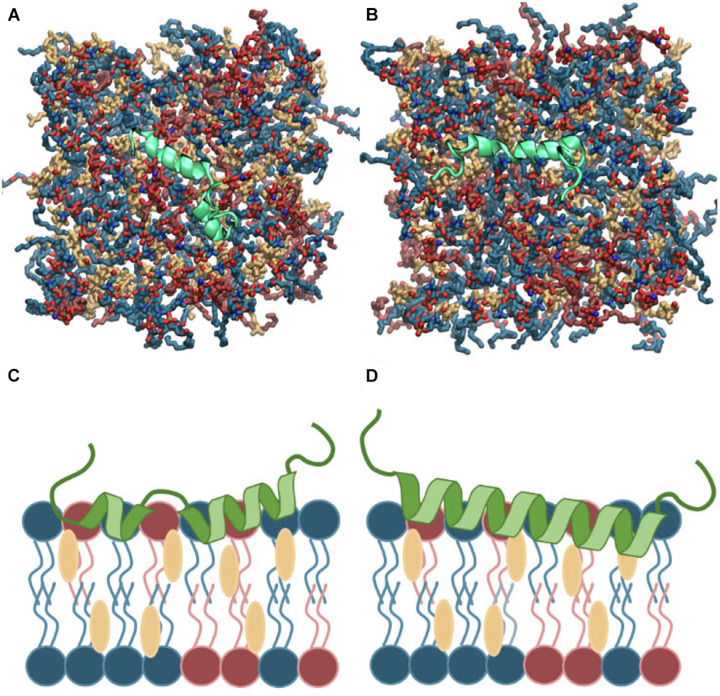
**(A,B)** Top view of the atomistic starting structures of kinked and straight IAPP peptides in the membrane bound helical conformation. Water and ions are not shown. **(C,D)** Sketches of the membrane bound state of kinked and straight IAPP peptides shown from the side. The peptide is illustrated in green and DOPC, DOPS, and CHOL are shown in blue, red, and yellow, respectively.

**FIGURE 3:**
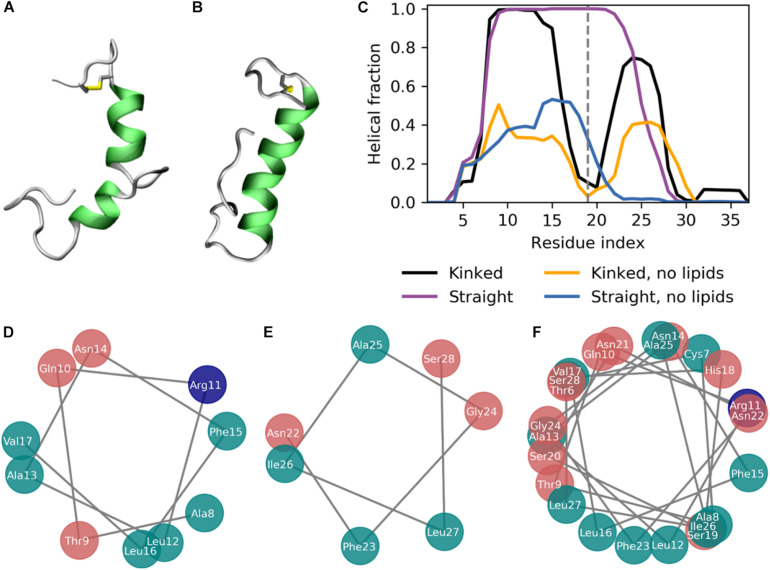
The secondary structure of IAPP in the kinked or straight conformation. **(A,B)** Simulation structures of a kinked (based on PDB-ID 2L86) and a straight peptide (based on PDB-ID 2KB8) with the helical residues colored green. **(C)** Fraction of combined simulation time with each residue in a helical conformation for the kinked and straight peptides in the presence, with and without the presence of lipid membranes. **(D)** Helical wheel of the N-terminal helix of the kinked peptides. **(E)** Helical wheel of the C-terminal helix of the kinked peptides. **(F)** Helical wheel of the straight helix of the straight peptides.

Islet amyloid polypeptide is expected to be helical in the membrane bound state (Jayasinghe and Langen, 2005; Knight et al., 2006; Engel, 2009; Khemtémourian et al., 2011; Lee et al., 2012). As for most other amphiphilic helices, crossing the lipid headgroup region involves an energy-barrier, and simulating the binding is therefore computationally challenging (Ben-Tal et al., 1996). Coarse-grained MD simulations of a single IAPP peptide placed above lipid bilayers of each of the chosen bilayer compositions were performed initially to position IAPP at the membrane surface, while maintaining the helical conformation (Supplementary Figure 1A). Within 0.5 μs the peptides were bound to the bilayer, with the amphiphilic helices in the hydrophobic/hydrophilic interface of the bilayer (Supplementary Figure 1B). Crossing the headgroup region is a complex process, but in the coarse grained simulation the process is simplified and the amphiphilic helices can quickly cross this barrier and be positioned at the hydrophobic/hydrophilic interface. The membrane bound structures were subsequently converted to atomistic structures using the Martini Backward script (Wassenaar et al., 2014). To increase the sampling, two starting conformations of the peptides were applied; a straight conformation with a helical conformation spanning from residue 7 to 28, and a kinked conformation with residues 7 to 18 and residues 23 to 28, respectively, forming each an α-helix, with a turn between the two helical regions (as sketched in Figures 2, 3). Both of these starting conformations are based on micelle bound structural ensembles of IAPP from NMR experiments (Patil et al., 2009; Nanga et al., 2011). This procedure was executed for different combinations of the lipids: DOPC, DOPS, and CHOL. Four repeats of 1 μs were produced for each of four membrane compositions: DOPC, DOPC/CHOL, DOPC/DOPS, and DOPC/DOPS/CHOL; and for each of the two initial peptide conformations (Straight/Kinked). An overview of the atomistic simulations can be found in Table 1. The membrane compositions are chosen to compare well with experimental data from the literature (Zhang et al., 2017). Examples of the DOPC/DOPS/CHOL setup is illustrated in Figure 2.

**TABLE 1:** Simulation overview of atomistic simulations with various membrane composition including DOPC, DOPS, and CHOL.

**Membrane composition**	**Lipid ratio**	**Helix conformation**	**Number of repeats**	**Simulation time**
–	–	Kinked	3	1 μs
		Straight	3	1 μs
DOPC	100%	–	1	1 μs
		Kinked	4	1 μs
		Straight	4	1 μs
DOPC:DOPS	70 – 30%	–	1	1 μs
		Kinked	4	1 μs
		Straight	4	1 μs
DOPC:CHOL	60 – 40%	–	1	1 μs
		Kinked	4	1 μs
		Straight	4	1 μs
DOPC:DOPS:CHOL	45 – 15 – 40%	–	1	1 μs
		Kinked	4	1 μs
		Straight	4	1 μs

### Coarse-Grained Simulations

The coarse-grained simulations were performed in Gromacs 2016.3 (Abraham et al., 2015) with the polarizable Martini 2.2P forcefield for protein and water (Yesylevskyy et al., 2010) and the lipids and ions modeled using the MARTINI 2.0 forcefield (Rzepiela et al., 2011; de Jong et al., 2013). The systems were built using the *Insane* script (Wassenaar et al., 2015), which can build lipid bilayers with the lipids randomly placed in each leaflet in the specified ratio and placing a peptide relative to the bilayer. The peptides were placed with the center of mass 40 Å from the membrane center and in a random orientation in each simulation repeat. The systems were minimized using steepest descent optimization, followed by a 50 ns equilibration with the number of particles, pressure, and temperature (NPT ensemble) kept constant, and with the protein constrained. The protein was released and a production run of 0.5 μs was performed in the NPT ensemble. The secondary structure of IAPP was restrained to the secondary structure of the starting structure, as is common practice in the MARTINI 2.0 forcefield (Rzepiela et al., 2011; de Jong et al., 2013). The flexible ends and kink region of IAPP were not constrained during the membrane binding simulations.

The leap-frog integrator (Hockney et al., 1974) was used with a timestep of 20 fs. During the NPT equilibration the Berendsen pressure coupling (Berendsen et al., 1984) was used with a time-constant of τ_p_ of 12 ps and reference pressure of 1 bar. The pressure coupling was applied semi-isotropically (x-y and z decoupled), and with an isothermal compressibility of 310^−4^bar^−1^ for the system. In the production run the pressure was controlled with the Parrinello-Rahman barostat (Parrinello and Rahman, 1981) with a τ_p_ of 24 ps, a compressibility of 310^−4^bar^−1^ and a reference pressure of 1 atm. The velocity rescaling algorithm was used to keep the temperature constant with a coupling constant τ_t_ of 1 ps. The coulomb interactions were treated using a reaction-field,(Tironi et al., 1995) with a cutoff of 1.1 nm and a relative electrostatic screening of 2.5 (de Jong et al., 2016). The van der Waals interactions are cut-off at 1.1 nm using a potential shift cutoff-scheme.

### Atomistic Simulations

The atomistic simulations were performed in Gromacs 2016.3 (Abraham et al., 2015) which uses the leap-frog integrator (Hockney et al., 1974). The lipids, water, and ions were modeled using the CHARMM36 force field (Klauda et al., 2010), and proteins were modeled using the CHARMM36m force field (Huang et al., 2016), a recently developed force field with improved parameters for simulating intrinsically disordered peptides and the recommended force field for simulations of proteins and lipids from the CHARMM force field developers (Huang et al., 2016). To obtain a 2 fs timestep, all bonds to hydrogens are constraint using the LINCS algorithm (Hess et al., 1997). All histidine was modeled as the neutral δ-tautomer, which is known to allow binding of IAPP to the bilayer surface (Skeby et al., 2016).

The membrane bound structures returned from the Martini *backward* script (Wassenaar et al., 2014), were first minimized using the steepest descent algorithm with a maximum of 5,000 steps. The initial velocities were assigned randomly from a Maxwell-Boltzmann distribution. Then a 0.1 ns equilibration was performed in a canonical ensemble with number of particles, volume, and temperature constant (NVT ensemble). Next, a 3 ns equilibration was performed in the NPT ensemble. Finally, a 1 μs simulation production run was performed, also in the NPT ensemble.

The electrostatic interactions were calculated using Particle Mesh Ewald (Essmann et al., 1995) with a grid spacing of 0.1 nm and a short-range cutoff of 1.2 nm, using the Verlet cutoff-scheme. The van der Waals interactions were calculated with a force-switch modification (Steinbach and Brooks, 1994) that switch off the interaction between 1.0 and 1.2 nm. The temperature was controlled using the Nose-Hoover thermostat (Hoover, 1985), with a time constant of 1 ps, to keep the temperature at a constant temperature of 310K. A pressure of 1 atm was held using the Parrinello-Rahman pressure coupling (Parrinello and Rahman, 1981), with a time-constant of 5 ps, applied uniformly in the x-y dimension and separate in the z-dimension.

## Results

A series of simulations of membranes with DOPC, DOPS, and CHOL was performed to investigate the interactions between IAPP and the lipid components of the membrane and to study the membrane bound structure of IAPP and how it depends on the bilayer composition. The simulations are summarized in Table 1 of the methods section.

### The Helical Conformations Are Stabilized at the Membrane Interface

The simulations were initiated in the α-helical membrane bound state. The initial structure of the peptides in this study is helical peptides with a kink (Figure 3A) or with a single straight helix (Figure 3B). It can be seen from Figure 3C, that the helicity is stabilized by the membrane, likely due to the amphiphilic nature of the helices, with the hydrophobic residues buried in the membrane and the hydrophilic residues exposed to the solvent. The amphiphilicity of the peptide can be seen from the orientation of the hydrophobic and hydrophilic residues in the helical wheels in Figures 3D–F generated from a projection of the Cα atoms on the plane perpendicular to the helix axes. The residues Ala8, Leu12, Phe15, Leu16, Phe23, Ile26, and Leu27 are the hydrophobic residues of the helices, that are oriented toward the interior of the membrane. Without the presence of a lipid membrane, the α-helix is not stable, and from the helical fractions in Figure 3C it can be seen that the helices are unfolded most of the time.

The helicity fraction is described as the fraction of the accumulated simulation time each residue is helical (Figure 3B). For membrane inserted peptides, the energy barrier for conformational changes is very high, and is thus an event that occurs at a relatively long timescale (Ulmschneider and Ulmschneider, 2018), and therefore the helicity of IAPP starting in the kinked or straight will be analyzed separately. For the peptides starting in the kinked conformation, the helical fraction in the N-terminus peaks from Cys7 to Leu16, with decreasing fractions toward each end of the helix. In the C-terminus the helicity peaks from residue Asn21 to Ile28 (Figure 2C). The helical fraction of the N-terminus peaks at 1, meaning that some central residues are not seen to unfold at any point during the simulations. The C-terminal peaks at 0.8, indicating that all residues are unfolded at some point in the simulation series. The helicity varies less for the straight peptides than the kinked peptides. From the helicity fraction of the straight peptides in Figure 3B it can be seen that the helicity peaks from residue Asn3 to Ser28, with residues Thr9 to Phe23 helical throughout all simulation repeats. In the kinked peptides, the turn connecting the two helical segments consist of the 18-HSSNN-22 fragment, these are all hydrophilic residues and can therefore not contribute to the hydrophobic side of the helix. However, the turn region has been proposed to be important for the oligomerization of IAPP. Especially His18 and the charge state of this residue is found to be important for interactions between the IAPPs (Abedini and Raleigh, 2005; Christensen et al., 2017). In the straight peptides the kink region is tackled by having Ser20 and Ser19 on the hydrophobic side of the helix, oriented toward the sides of the helix, possibly allowing the hydrophilic sidechains to interact with the hydrophilic headgroups (Figure 3F).

**FIGURE 4:**
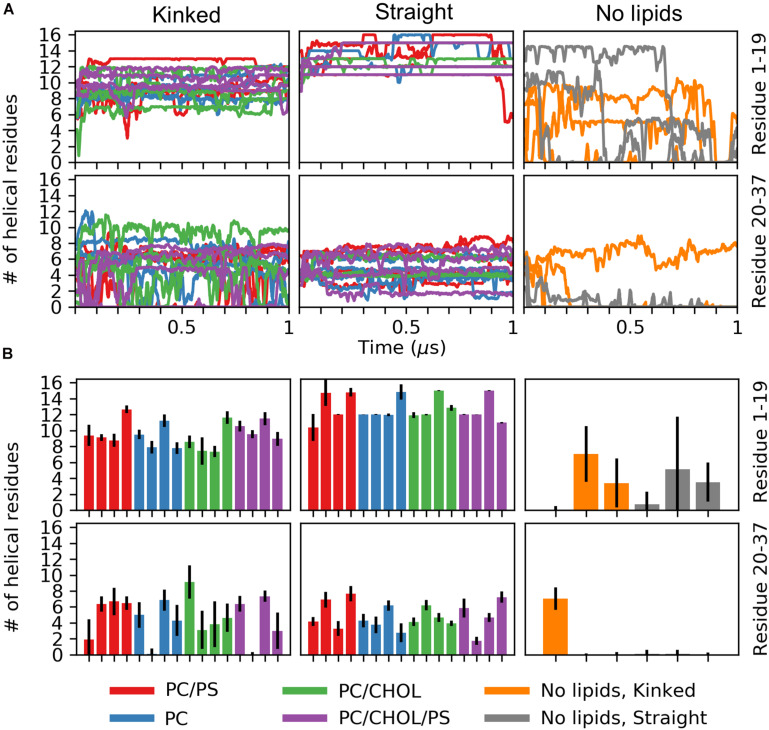
Number of helical residues for each series of simulation (PC in blue, PC/CHOL in green, PC/PS in red, and PC/PS/CHOL in purple). The number of helical residues is plotted separately for the N-terminus (residue 1–19, first row) and the C-terminal (residue 20–37, second row) and separated for the peptides starting in the kinked conformation and the straight conformation. **(A)** The development of the number of residues in a helical conformation in time. **(B)** Mean and standard deviation of the number of helical residues in each of the simulation repeat.

The number of helical residues in the course of the simulations is shown in Figure 4A, separated for the N-terminal residues Lys1 to Ser19 and the C-terminal residues Ser20 to Tyr37, and when a lipid membrane is present. Figure 4B shows mean and standard deviation of the number of helical residues for the last 0.5 μs of the simulations. For the peptides initiated in a kinked conformations, the number of helical residues in the N-terminus remains around 10 and shows very little fluctuation in the simulations. The largest fluctuations in the helicity observed is in the C-terminus, as seen from Figure 4A only around five residues remain helical, and in some simulations the C-terminal helix completely unfolds. The peptides starting in the straight conformation have around 13 and 5 helical residues in the N-terminus and C-terminus, respectively. Both parts of the straight helix seems to be more stable than the two helical segments of the kinked peptides, as seen from the small error bars for the straight peptides in Figure 4B. It can be speculated that the stability of the straight peptides is most likely due to the fact that it has fewer helix ends where unfolding can occur, and an increase in intramolecular interactions arising from the secondary structure. There are no obvious differences in the stability of the helicity depending on the membrane composition. In the simulations of IAPP in solvent, with no lipid bilayer in the simulations, the helicity is less stable as seen from Figures 4A,B. The N-terminal helix is unfolded in all repeats and only in a single repeat does the C-terminal part of the straight helix remain helical.

### Insertion of Peptide on the Membrane Is Affected by the Membrane Lipids and the Peptide Conformation

**FIGURE 5:**
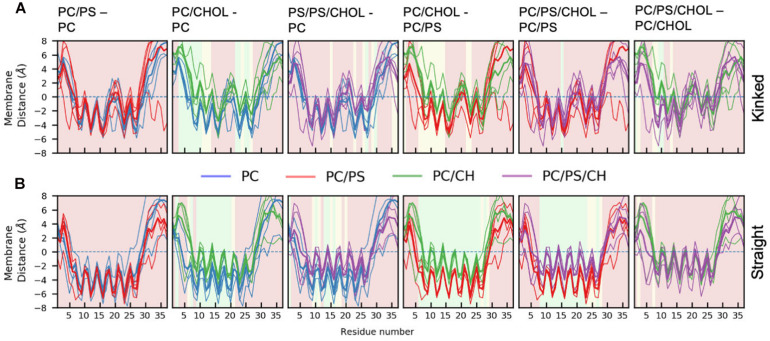
Position of Cα of the peptide relative to the phosphate plane for. **(A)** The kinked conformation **(B)** straight conformation. Each panel shows the average distance between the IAPP Cα and the x-y plane spanned at the average z-coordinate of the phospholipid phosphates (indicated with a dashed line). The lines are colored based on the composition of the lipid membrane in the simulations. The average for the individual simulations are shown in thin lines and the common average in a thicker line. For the sake of comparison, two systems are shown simultaneously. The background color of the plots indicates whether the position on the membrane is significantly different between the two simulation series (green: *p* ≤ 0.05, yellow: 0.05 < *p* < 0.10, red: *p* > 0.10, as explained below).

Figure 5 shows the average distance between each residue and the x/y-plane as measured as the average z-coordinate of the phospholipid phosphates, with z being the direction of the bilayer normal; the plane is used as an indication of the hydrophobic/hydrophilic interface. The simulation series are compared in pairs to show the effect of adding additional lipid types to the simulation system. In all simulations the hydrophilic residues of the helix are pointing away from the membrane while the hydrophobic residues are mostly pointing toward the membrane, giving rise to a zigzag-shaped curve. Most of the helical residues are placed below the hydrophobic/hydrophilic interface, indicating that the peptides are inserted in the membrane. Generally the highest variation in the membrane distance is seen in the non-helical region (residue 1 to 7 and residue 27 to 37), since these parts of the peptide are not usually inserted into the membrane.

**FIGURE 6:**
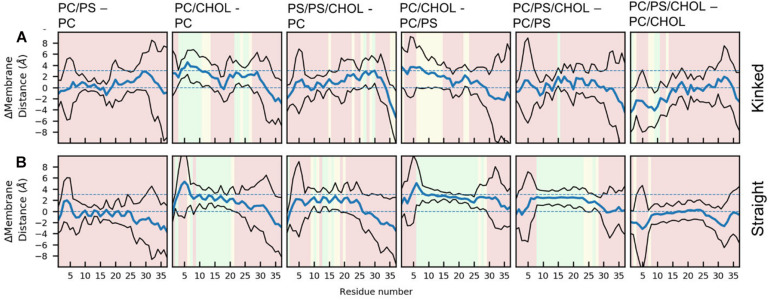
Each panel shows the differences between the peptide insertion in the **(A)** kinked and the **(B)** straight IAPP. The mean and standard deviation is indicated with a blue and black lines, respectively. The background color of the plots indicates the significance of the difference (green: *p* ≤ 0.05, yellow: 0.05 < *p* < 0.10, red: *p* > 0.10).

The difference in the mean residue-membrane distances (Δmembrane distance) between the two simulation series in question is shown in Figure 6. The background color of the plots is an indication of whether the Δmembrane distance is significantly different from 0, using the *T*-test for comparing the means of two simulation series. The difference between pure PC and PC/PS membranes has a negligible effect on the membrane distance in both the kinked and the straight conformation (Figures 6A,B), as seen from a Δmembrane distance close to 0. This indicates that the membrane bound state of IAPP is well-defined in these simple phospholipid membranes. Addition of CHOL to a DOPC membrane increases the distance between the peptides and the membrane interface, and most significantly in the helical regions, as seen from an increase in Δmembrane distance of up to 4 Å in Figures 6A,B. The same is observed when comparing the membrane distance of a PC/PS membrane to a PC/CHOL membrane (Figures 6A,B). Interestingly, the peptides starting in the kinked conformation bound to a PC/PS and a PC/PS/CHOL membrane have the similar distance to the membrane interface (Figure 6A), this indicates that PS lipids keep the helices closer to the membrane, and thus counteracts the effect of CHOL. This effect is, however, not seen for the straight peptides, where the addition of CHOL still significantly increases the membrane distances compared to PC/PS membranes (Figure 6B).

**FIGURE 7:**
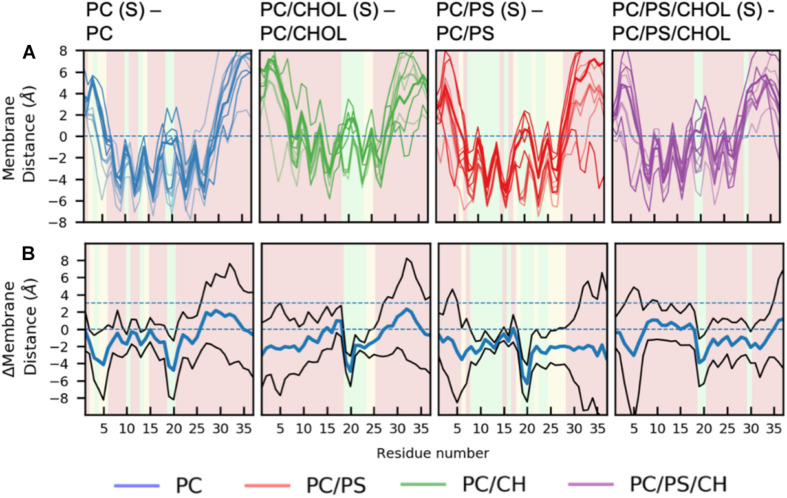
Comparison of membrane positions of straight and kinked IAPP relative to the membrane. **(A)** Position of Cα of the peptide relative to the phosphate plane, colored as in Figure 5. **(B)** Differences between the peptide insertion in the kinked and the straight IAPP, colored as in Figure 6.

There are general differences between the position of the kinked and the straight peptides. Comparing Figures 5A,B, it can be observed that the top of the helical segment of the straight peptides is positioned about 2 Å below the hydrophobic/hydrophilic interface in a pure phospholipid membrane, and the top of kinked helices in corresponding membranes are positioned at the level of the hydrophobic/hydrophilic interface. The membrane positions and the insertion for the straight and the kinked peptides are compared in Figures 7A,B. Not surprisingly, the turn region (residue 19–22) is positioned significantly deeper in all simulations with straight peptides when comparing to the kinked peptides. In the pure phospholipid membranes (pure DOPC or DOPC/DOPS), the conformation affects the position of the helical segments. In the DOPC membrane the N-terminal helix is inserted about 2 Å deeper for the peptide in the straight conformation, and most significantly for residues Gln10 and Asn14 (Figure 7B). In the DOPC/DOPS membranes both parts of the helix are inserted significantly deeper in the membrane by about 2 Å (Figure 7B). In the CHOL containing membranes there is no significant difference in the membrane distance, other than in the kink region, as seen in Figure 7B.

### Cholesterol Increases the Order and Thickness of Lipid Bilayers

**FIGURE 8:**
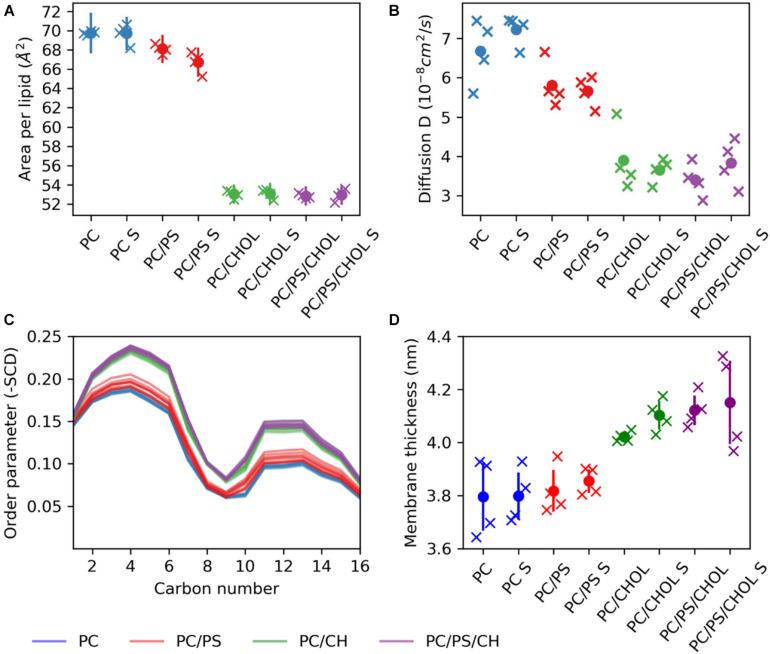
Membrane properties. **(A)** Area per lipid. **(B)** Diffusion coefficient. **(C)** Order parameter **(D)** Membrane thickness. The properties are shown for each simulation series (each individual simulation marked by an X), with S indicating the simulation series with IAPP starting in the straight conformation.

The difference in the position of IAPP on the membrane upon the addition of CHOL and/or DOPS can either be due to changes in the membrane fluidity or arise from direct interactions with the lipids. With the addition of CHOL to the lipid bilayers the order and rigidity is expected to increase (Gracià et al., 2010). The area per lipid was calculated using the *Membrainy* tool (Carr and MacPhee, 2015). From Figure 8A it can be seen that in the simulations with CHOL, the area per lipid is decreased from around 70Å^2^ to ∼53Å^2^. Changing from a composition of DOPC to DOPC/DOPS decreases the area per lipid slightly from around 70Å^2^ to around 67Å^2^. The lipid diffusion is also affected by the addition of CHOL (Figure 8B), in the DOPC bilayers the lipid diffusion is about 7 ⋅ 10^−8^cm^2^/s, whereas in a DOPC/CHOL bilayer the diffusion has dropped to half, to about 3.5⋅10^−8^cm^2^/s. The diffusion in a DOPC/DOPS bilayer is about 5.5⋅10^−8^cm^2^/s, slightly less than for a pure DOPC bilayer. The diffusion coefficients were calculated using the *gmx msd* tool of GROMACS 2018.1 (Abraham et al., 2015). The same pattern is observed for the order parameter (Figure 8C): CHOL increases the order parameter, and DOPC/DOPS membranes are also slightly more ordered than DOPC membranes. The order parameters were also calculated using the *Membrainy* tool (Carr and MacPhee, 2015). Another sign of the membranes with CHOL bring more ordered is reflected in the membrane thickness (Figure 8D), which is increased from around 3.8 nm to around 4.1 nm when adding CHOL. The membrane thickness was calculated from the distance between the peaks of the phospholipid phosphate density of each leaflet.

For the simulations with the straight peptides, the increased membrane distance in the presence of CHOL correlates with the increased membrane thickness and order. This effect is, however, not seen in the simulations with the kinked peptide, where the presence of DOPS lipids seems to diminish the effect of CHOL on the positioning of the helix on the membrane.

### Anionic Lipids Are Enriched in the Vicinity of IAPP and Cholesterol Is Depleted

**FIGURE 9:**
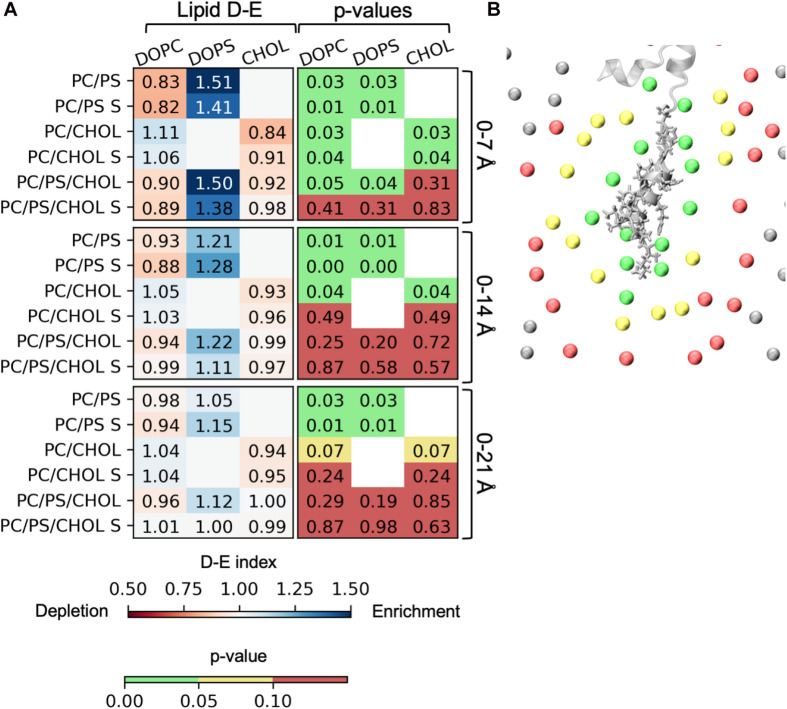
Depletion enrichment index (D-E index) of lipids around the peptide. **(A)** The panels represent the D-E index of the lipids within 7, 14, and 21 Å. The first column shows the values of the D-E index, colored on a scale from 0.5 to 1.5. The second column shows the *p*-values. The statistical significance was evaluated using the single sample *T*-test, with 1 as the null-hypothesis, indicating that there is no enrichment or depletion of a given lipid type around the peptide. **(B)** The phospholipid phosphates within 7 Å (green), 14 Å (yellow), and 21 Å (red) of IAPP_1__–__19_. The D-E index is calculated for each simulation series, and the simulation series with IAPP starting in the straight conformation are denoted with an “S.”

The lipid depletion-enrichment index (D-E index) is calculated to investigate local concentration variations of the lipids surrounding the peptide (Corradi et al., 2018). The analysis is based on the method developed by Corradi et al. (2018) and (Corradi et al., 2018). The first panel in Figure 9A shows the D-E index of lipids within the first lipid shell surrounding the membrane bound peptide (lipids within 7 Å of the IAPP_1__–__19_). For the systems with DOPC and DOPS lipid there is a significant depletion of DOPC and a significant enrichment DOPS within the at all the measured distances (7, 14, and 21 Å), the effect is, however, decreasing with the distance. The distances 7, 14, and 21 Å, were used in the original paper by Corradi et al. (2018) and represent the three nearest lipid shells around IAPP_1__–__19_, as illustrated in Figure 9B. The enrichment is most likely due to favorable interactions between the cationic residues of IAPP and the anionic groups of the PS headgroup. In the systems with DOPC and CHOL, there is a slight depletion of CHOL within the first shell around the peptide. The depletion of CHOL is independent of DOPS, and therefore indicates a preference for interactions between the helix and phospholipids rather than CHOL, as seen by the values below 1 in the CHOL column for the 0–7 Å range. More variation is seen in the three component system with both DOPC, DOPS, and CHOL, the DE-indices are less significant, probably due to the increased possibility of variation, and thus more sampling is required. However, the enrichment of DOPS in the inner shell is significant for the systems starting with the peptide in a kinked conformation.

### Flexibility in the N-terminal Loop Shows Two Dominant Orientations

Since the effect of the lipids on the position of IAPP can not be explained completely by the physical properties of the membrane, a structural clustering is performed to investigate the specific interactions between the lipids and IAPP. In order to perform a structural clustering of IAPP and the membrane lipids, the conformational flexibility of IAPP has to be taken into account. The largest flexibility is in (a) the N-terminal loop (residue Lys1 to Cys7), (b) in the C-terminus after the C-terminal helix from residue Ser28 to Tyr37, and (c) in the kink region of the peptides between the N-terminal and C-terminal helix; the kink usually includes residue His18 to Phe23, and allows for variation in the angle between the two helices.

**FIGURE 10:**
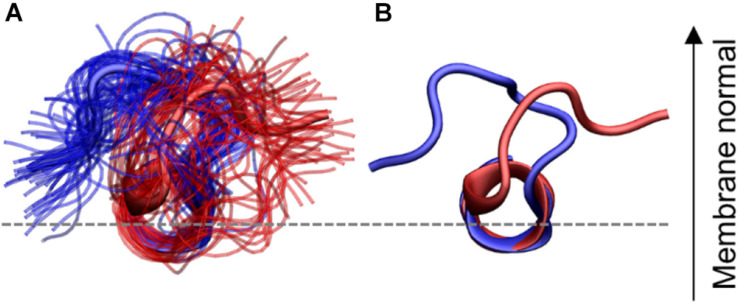
N-terminal loop orientations, structures with loop orientation 1 (O1) and 2 (O2) are shown in blue and red, respectively. **(A)** Ensemble of structures from the two largest clusters **(B)** central structure from each of the two orientations. The C-terminal region is hidden for clarity.

A geometric clustering was performed to better understand the flexibility in the N-terminal loop (More details are available in the Supplementary Material). It was found to be oriented in one of two ways for most of the simulation time. Either the N-terminal loop is pointing to the left or the right side compared to the helix, when viewing from the N-terminus, as illustrated in Figure 10: These two orientations collect 72.8% of the conformations sampled by the peptides (38.0 and 34.8%, for orientation 1 and 2, respectively) the additional clusters contained far fewer structures (Supplementary Figure 2). In the individual simulations the peptide does not sample both orientations equally. Figures of the distribution of the orientations for each simulation repeat are found in the SI along with calculations of the median structure for each repeat. It is seen from the graphs that when CHOL is present IAPP orientation 1 (O1) is slightly favored over orientation 2 (O2).

The effect of the orientation of the N-terminal loop might seem insignificant, however, the N-terminal loop contains two of the charges and is therefore essential for the interaction with the phospholipids. In addition, Cys2-Cys7 disulfide bridge in the N-terminal loop has also been proposed to decrease the propensity of IAPP to aggregate (Milardi et al., 2008). The importance of the N-terminal orientation will become apparent in the following analysis, about the specific interactions between IAPP and the membrane lipids.

### Certain Regions of the N-terminal Helix Favor Lipid Interactions

**FIGURE 11:**
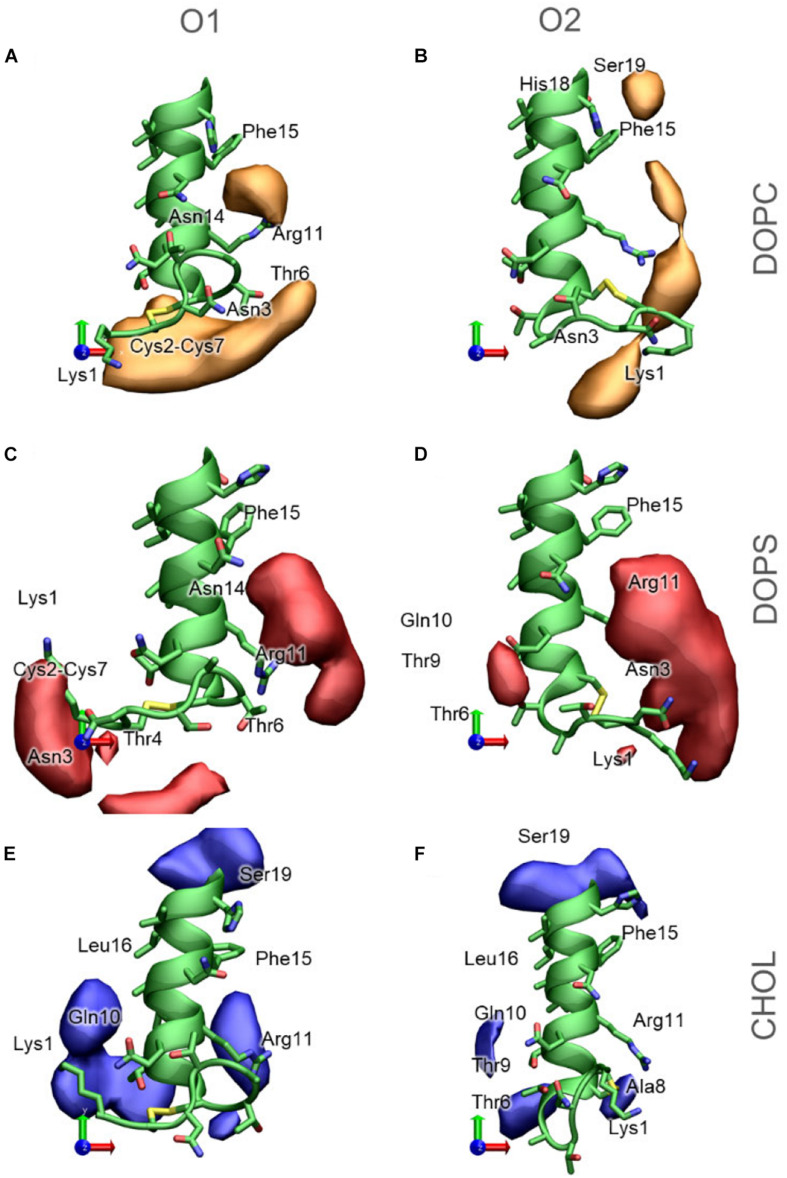
3D Occupancy maps of the lipids around IAPP_1__−__19_, depending on the orientation of the N-terminal loop **(A)** DOPC around IAPP_*O1*_ **(B)** DOPC around IAPP_*O2*_ **(C)** DOPS around IAPP_*O1*_ **(D)** DOPS around IAPP_*O2*_ **(E)** CHOL around IAPP_*O1*_ **(F)** CHOL around IAPP_*O2*_. IAPP_1__−__19_ is shown in green, and selected residues are labeled.

The 3D occupancy maps in Figure 11 reveals the volumes around IAPP_1__–__19_ with a high density of DOPC, DOPS and CHOL in the simulations (with the peptide aligned in the helical segment IAPP_7__–__16_). The analysis is repeated for IAPP with the N-terminal loop in each of the two orientations. The occupancy maps can be seen from other angles in Supplementary Figure 4.

DOPC is the lipid present at the highest concentration in the bilayer in all simulations. The density map in Figure 11A indicates that DOPC preferentially interacts with IAPP_*O1*_ in two regions around the peptide; at the N-terminal of the peptide, where DOPC interacts with the loop region (Lys1 – Cys7). Seen from above (Figure 11A) DOPC also preferentially interacts with the right side of the N-terminal helix, on this side it can interact with Arg11, Phe15, and Asn14. At the kink region, DOPC has a preferential interaction near Phe15, His18, and Ser19. The interaction volumes of DOPC are very dependent on the orientation of the N-terminal loop; DOPC interacts mainly with IAPP_*O2*_ in the loop region around Lys1, between Lys1 and Arg11, between Arg11 and Phe15, and near His18 and Phe15 (Figure 11B). In both of the orientations of the N-terminal loop, DOPC shows a preference toward binding to the right side of the helix (Figures 11A,B).

DOPS is negatively charged, with two negative charges and one positive charge in the head group, and therefore preferentially interacts with the cationic groups of IAPP. In IAPP_*O1*_, DOPS interacts near the N-terminus and Lys1 or between Arg11, Phe15, and Asn14 (Figure 11C). IAPP_*O2*_ has the positive charges of Lys1 and the N-terminus on the same side as Arg11, which makes it favorable for DOPS to be positioned in the cleft between these residues, to the right side of the helix (Figure 11D).

Cholesterol is much more hydrophobic than DOPC and DOPS, and is therefore less prone to interactions with the hydrophilic face of the amphipathic helix. However, CHOL has a hydroxyl group that preferentially interacts with the hydrophilic groups near the interface, such as exposed backbone atoms and residue sidechains at the helix ends (Figures 11E,F). The interaction volumes of CHOL are mostly positioned below the helix, in the vicinity of the hydrophobic residues Leu12, Leu16, and Phe15. The interactions are unspecific, more evenly distributed around the IAPP than the phospholipids, and seem to be less dependent on the orientation of the N-terminal loop and Lys1 (Figures 11E,F).

### The Binding Modes of the Lipids May Be Related to the Insertion of the Peptide

For a more detailed description of preferred binding sites of the membrane lipids to IAPP we have performed a distance based clustering of the helix-lipid pairs (details available in Supplementary Material). It was observed from the 3D Occupancy maps, that CHOL interacts differently with IAPP_1__–__19_ than the phospholipids, and these will therefore be presented separately. The following analysis is based on a collection of all the IAPP-lipid pairs in all structures with the N-terminal loop in either O1 or O2.

**FIGURE 12:**
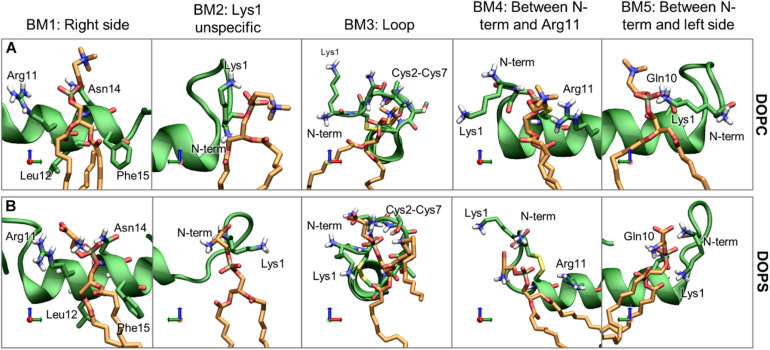
Representative structures of the phospholipid binding modes (BM1-5), for **(A)** DOPC and **(B)** DOPS. IAPP is shown in green, with green carbons, and the lipids are shown with orange carbons.

For the phospholipids, the dominant interactions are very dependent on hydrogen bonding and salt bridge interactions between the lipids headgroup and the hydrophilic residues. The most frequently occurring binding modes (BMs) of DOPC and DOPS are illustrated with representative structure in Figure 12, and the relative frequencies are shown in Supplementary Table 1. The five largest cluster will be discussed here, as they constitute the vast majority of the interactions.

*Binding mode 1* (BM1) This is the most prominent lipid binding mode placed on the right side of the helix, with the lipid binding in a pocket between Arg11 and Asn14 and the tails between Leu12 and Phe15. This binding mode is independent of the orientation of the N-terminal loop and the binding mode is observed for both DOPC and DOPS lipids. *Binding mode 2* (BM2) is another prominent “binding mode.” It entails the unspecific salt-bridge interactions with Lys1, both DOPC and DOPS has a negative charge at the phospholipid phosphate which can interact with Lys1, the N-terminus, or bridging between the two. In the case of DOPS, the two negative charges can interact with Lys1 and the N-terminus simultaneously, thus making this interaction very favorable for DOPS lipids, as seen from the relatively high occurrence of this binding mode for DOPS lipids compared to DOPC lipids. Independent of the orientation of the N-terminal loop, it is a common interaction point for the phospholipids. In *Binding mode 3* (BM3) the N-terminal loop between Cys2 and Cys7 consists of several hydrophilic residues (Asn3, Thr4, and Thr6), and it has exposed backbone atoms, making it a good interaction point for the hydrophilic headgroups of both DOPC and DOPS. In O2 of *Binding mode 4* (BM4) the N-terminus and Lys1 is pointing toward the right side of the helix. In this orientation the phospholipid can interact with Lys1 (or the N-terminus) and Arg11 simultaneously. *Binding mode 5* (BM5) only occurs for the loop in O1, where the phospholipids can bind between Gln10 and Lys1 (or the N-terminus).

In addition to the binding modes presented here, less frequently occurring binding encounters are found, e.g., some binding modes interacting with the kinked region, more details about these binding modes are available in Supplementary Material. The major binding modes presented here compares well with the 3D occupancy maps in Figure 8, however, the binding modes that involve the more flexible loop region and Lys1 might be under-represented in the occupancy maps.

It would be expected that the membrane composition affects the distribution and frequency of the lipid binding modes. The lipids could potentially be competing for the binding site, and the change in positioning of the helix in the presence of CHOL could affect the frequency of experiencing a given binding modes. The binding frequency of the binding modes could also be dependent on whether IAPP is straight or kinked. The fraction of each simulations series contributing to the binding modes of phospholipids and CHOL is shown in Supplementary Figures 7, 8, respectively. Few trends can be observed: For DOPC the contribution from each simulation is very heterogeneous across the simulation series, this indicates that the other lipids are not significantly competing with DOPC for any of the binding modes. In the interactions between DOPS and IAPP in O1, the straight peptides contributes most to BM1 and BM2, while the kinked peptides seem to provide better lipid interactions as in BM3 and BM5. None of these binding modes are in the kink-region, which indicates that the difference might arise from the difference in membrane insertion, which depends on the conformation of IAPP (as shown in Figure 7). There is a smaller fraction of the DOPS lipids interacting in BM4 in the presence of CHOL, which could indicate competing interactions.

**FIGURE 13:**
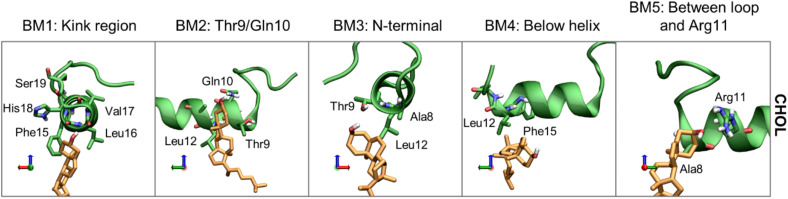
Representative structures of the CHOL binding modes (BM1-5). IAPP is shown in green, with green carbons, and the CHOL is shown with orange carbons.

Moving on to cholesterol, the 3D Occupancy maps show the interactions between the N-terminal helix and CHOL is less dependent on the orientation of the N-terminal loop. The interactions between CHOL and the N-terminal helix are primarily defined by hydrophobic interactions and hydrogen bonding of the hydroxyl group. The binding modes of CHOL are depicted in Figure 13, and their relative frequencies are listed in Supplementary Table 2.

*Binding mode 1* (BM1) of CHOL to the N-terminus of IAPP is at the kink region, this region has several hydrophilic residues (His18 and Ser19) and exposed backbone in the kinked peptides. At the kink region the hydrophobic residues Phe15, Leu16 and Val17 can interact with the hydrophobic rings of CHOL. Even for the straight peptides the kink region is an important interaction point (Supplementary Figure 8), due to interactions with these residues. *Binding mode 2* (BM2) is at the left side of the helix, near the N-terminal. In BM2, CHOL hydrogen bond to Thr9 or Gln10, and interact with the hydrophobic residues Leu12 and Ala13. In *binding mode 3* (BM3) CHOL interacts with the N-terminal helix end, most frequently interacting with Ala8, Thr9, and Leu12, and the exposed backbone atoms. *Binding mode 4* (BM4) is very unspecific, in this binding position CHOL interacts with the hydrophobic residues below the helix, most frequently Leu12 and Phe15. In *Binding mode 5* (BM5), CHOL interacts with the right side of the helix between the loop and Arg11, with hydrogen bonding to Arg11 and hydrophobic interactions with Ala8.

The relative occurrence of the CHOL binding modes can also be affected by the orientation of the N-terminal loop, the conformation of IAPP (straight or kinked), and the membrane composition. The relative contribution of each simulations series to the binding modes is shown in Supplementary Figure 8.

Binding mode 1 involves binding in the kink region, it is therefore expected to be affected by whether IAPP is in a straight or a kinked conformation. In the PC/CHOL membranes, BM1 is observed more frequently for the CHOL binding to IAPP in the kinked conformation than in the straight conformation, independent of the orientation of the N-terminal loop. In the PC/PS/CHOL membrane, this trend is not observed, and their relative binding frequency is independent of the conformation of IAPP, as seen in Supplementary Figure 8. This also indicates that the binding frequency of CHOL in BM1 on IAPP in the kinked conformation is reduced in the presence of DOPS (Supplementary Figure 8). Since DOPS does not have a strong tendency to interact with the kink region (As seen in Figure 11C), it is not likely that the effect is due to competitive interactions. In the system PC/CHOL S (straight conformation), PC/PS/CHOL, and PC/PS/CHOL S, IAPP is inserted deeper in the membrane than in the PC/CHOL systems, as shown in Figure 7. It could therefore be, that the change in the binding frequency of CHOL in BM1 is affected by the positioning of IAPP in the membrane, which could make binding in the kinked region less favorable.

Other than the variations in the binding frequency in BM1 there are no major variations across the systems. The binding modes are very evenly distributed around IAPP_1__–__19_, which was also observed from the 3D occupancy maps in Figure 11.

## Conclusion

Cholesterol is an important element of the lipid membrane, and it is often present as a sizeable fraction of the membrane lipids. CHOL affect the aggregation propensity and membrane effects of IAPP, however, the specific role of CHOL is unclear. This study investigates the effect of CHOL on the membrane bound state of IAPP.

The results presented here, confirm the stabilizing effect of lipid bilayers toward the helical structures of IAPP. Both the straight and the kinked conformations of IAPP were found to be stable on the membrane but unfolded in solution. The C-terminus was found to be less structured than the core membrane binding region in the N-terminal half of the peptide, which is in good agreement with previous results (Skeby et al., 2016). No significant variation of the helix stability was observed between the membrane compositions, but the straight helices were found to be more stable than the kinked helices, as seen from the secondary structure calculation shown in Figure 4.

It was found that the depth of membrane insertion of the amphipathic helices was affected by CHOL and whether IAPP was in a straight or a kinked conformation. CHOL generally results in a less deep insertion of the IAPP, than in pure phospholipid membranes, which can be explained by the increased ordering of lipids. A deep binding indicates a strong binding, and IAPP is observed to bind weaker to ordered membranes in experiments (Zhang et al., 2018). The present study has revealed that a membrane compositions of PC/PS/CHOL and a kinked conformation of IAPP, the PS lipids diminishes the effect of CHOL, and IAPP binds as deeply as in the pure phospholipid membranes, as seen from the membrane distance calculation in Figure 6. This concurs with the experimental results from Zhang et al. (2017) that CHOL in simple phospholipid membranes generally inhibits membrane induced amyloid formation of IAPP, and that the presence of PS lipids diminishes the inhibiting effect of CHOL. The insertion depth of IAPP was also affected by the conformation of IAPP, the straight conformation inserted significantly deeper than the kinked conformation, especially in the pure phospholipid membranes.

It was found that the membrane patch around IAPP_1__–__19_ was enriched with DOPS lipids and slightly depleted of CHOL, as seen from the D-E indices in Figure 9. The interactions between IAPP_1__–__19_ and the phospholipids is very dependent on the positively charged groups (N-terminal, Lys1, and Arg11). CHOL has a less well-defined interaction pattern, and it mostly interacts underneath the helix and with the kink region, as can be observed from the most frequent CHOL binding modes and 3D occupancy maps. It has been proposed from experiments that CHOL interacts directly with Phe15, which increases the binding affinity of IAPP to the membrane (Hao et al., 2018). It was proposed that CHOL interacted with a specific CHOL binding segment that involved a CARC motif that included Arg11, Phe15, and Val17, but this is not supported by our results. Phe15 is, however, found to be important for CHOL interaction, since Phe15 is centrally positioned in the N-terminal helix, and thus involved in many of the binding modes.

Based on these results we propose, that the effect of CHOL on the membrane bound state of IAPP and the membrane induced amyloid formation, is based on changes in insertion of IAPP due to changes in the physical properties of the membrane, rather than through direct interactions. We managed to observe significant changes in the insertion depth of IAPP in the presence of CHOL, and we found that PS can diminish the effect of CHOL. This provides a structural interpretation of the effect of CHOL on the membrane binding and the effect of the lipids on the aggregation rate.

This study is a step toward a deeper understanding of the role of CHOL in IAPP aggregation. Further studies, which take lipid raft formation into account need to be performed, but this topic is reserved for future work.

## Data Availability Statement

The raw data supporting the conclusions of this article will be made available by the authors, without undue reservation.

## Author Contributions

MC did the simulations and analysis. BS designed the project and supervised the work. All authors wrote the manuscript.

## Conflict of Interest

The authors declare that the research was conducted in the absence of any commercial or financial relationships that could be construed as a potential conflict of interest.
