# Supplementary material for: The Effect of Cholesterol on Membrane-Bound Islet Amyloid Polypeptide
Source: Front Mol Biosci. 2021 Apr 22;8:657946. doi: 10.3389/fmolb.2021.657946 (PMC8100463; doi:10.3389/fmolb.2021.657946)
Supplement: Supplementary file 1 [file Data_Sheet_1.pdf]

Supplementary Material

1 Supplementary Figures and Tables

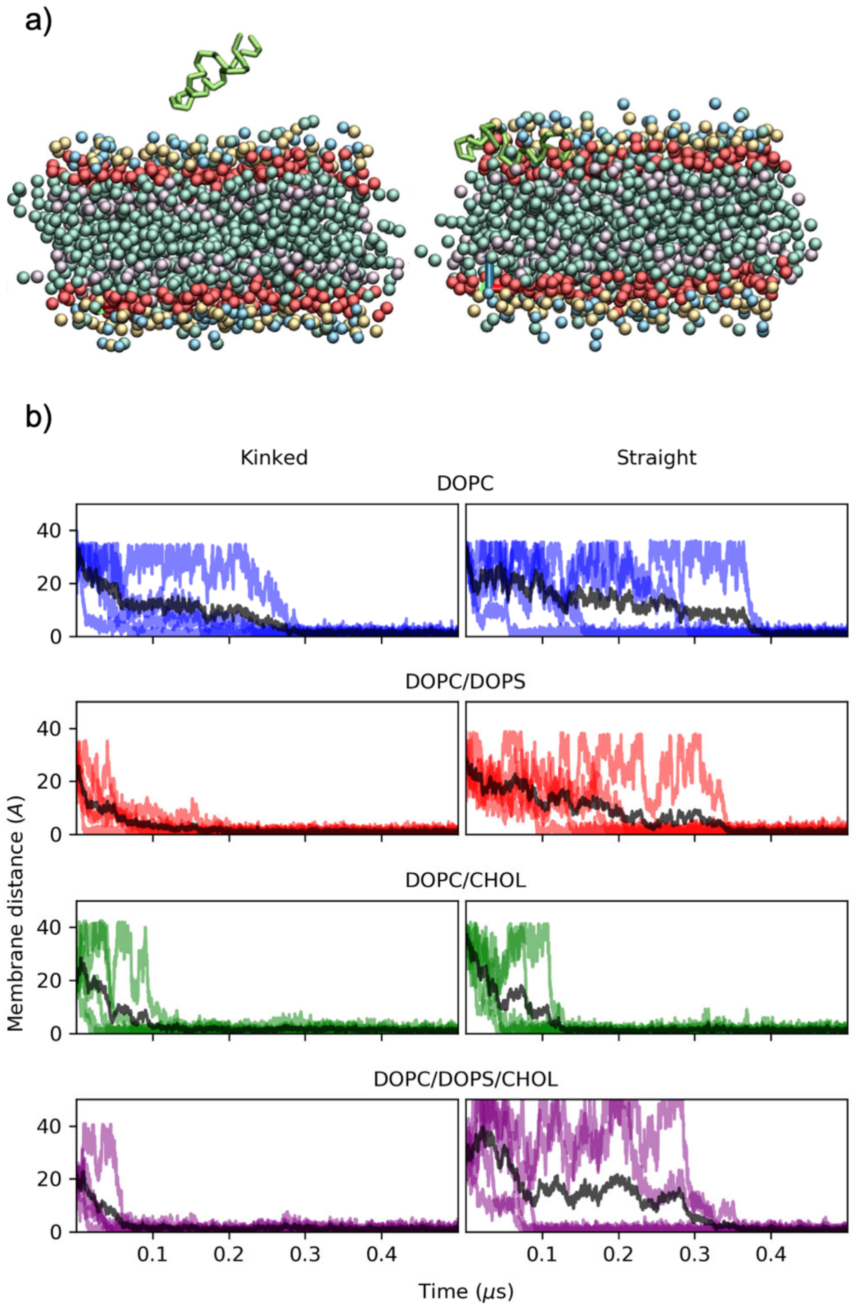

Supplementary Figure 1. a) starting structure (left) and membrane bound structure (right) of IAPP in an example of a coarse grained binding simulation. IAPP is shown in green and the membrane is colored based on the various MARTINI bead types b) Average distance between IAPP and the average position of the phospholipid phosphates in the nearest leaflet. Shown for each of the four membrane composition for each of the two IAPP conformations.

N-terminal clustering

The N-terminal was clustered based on the position of the atoms of the backbone of residue Lys1 to Asn14. The clustering module of Gromacs 2018.1[34] was used with the Gromos algorithm[49] and an RMSD cutoff of 0.35 nm. The frames where clustered for 0.2 ns, yielding a total of 5000 structures for each 1 µs simulation repeat, and a total of 160000 structures when combining all 4 repeats of all 8 systems. The two largest clusters contain 72.8 % of the structures, after which the subsequent clusters contributed with very few structures.

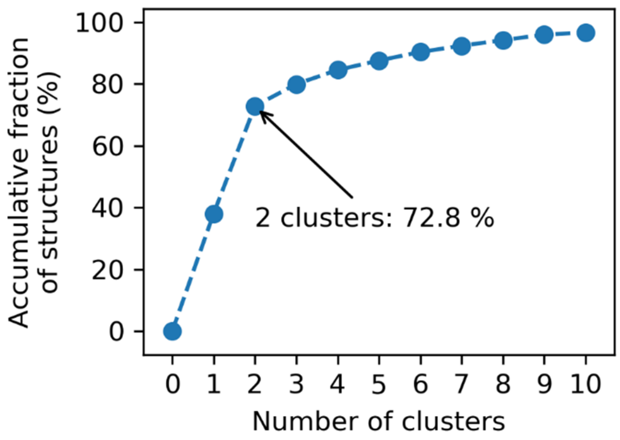

Supplementary Figure 2. Accumulative fraction of structures described by the clusters of N-terminal orientations for the 10 largest clusters.

The two largest clusters describes the orientation of the N-terminal loop and denoted IAPP orientation 1 ($IAPP_{O1}$) and orientation 2 ($IAPP_{O2}$). The contributions from each simulation are illustrated in Error! Reference source not found.**.** The simulations do not contribute to the two clusters equally, possibly due to insufficient sampling to observe many transitions between conformations. In a repeat of both the PC, PC/PS/CHOL, and PC/PS/CHOL:S simulation series, the N-terminal loop does not sample structures belonging two either of the two clusters. There seems to be a trend that the peptides on cholesterol containing membranes sample the $IAPP_{O1}$ more than $IAPP_{O2}$, however this could also be an artifact of insufficient sampling between conformations.

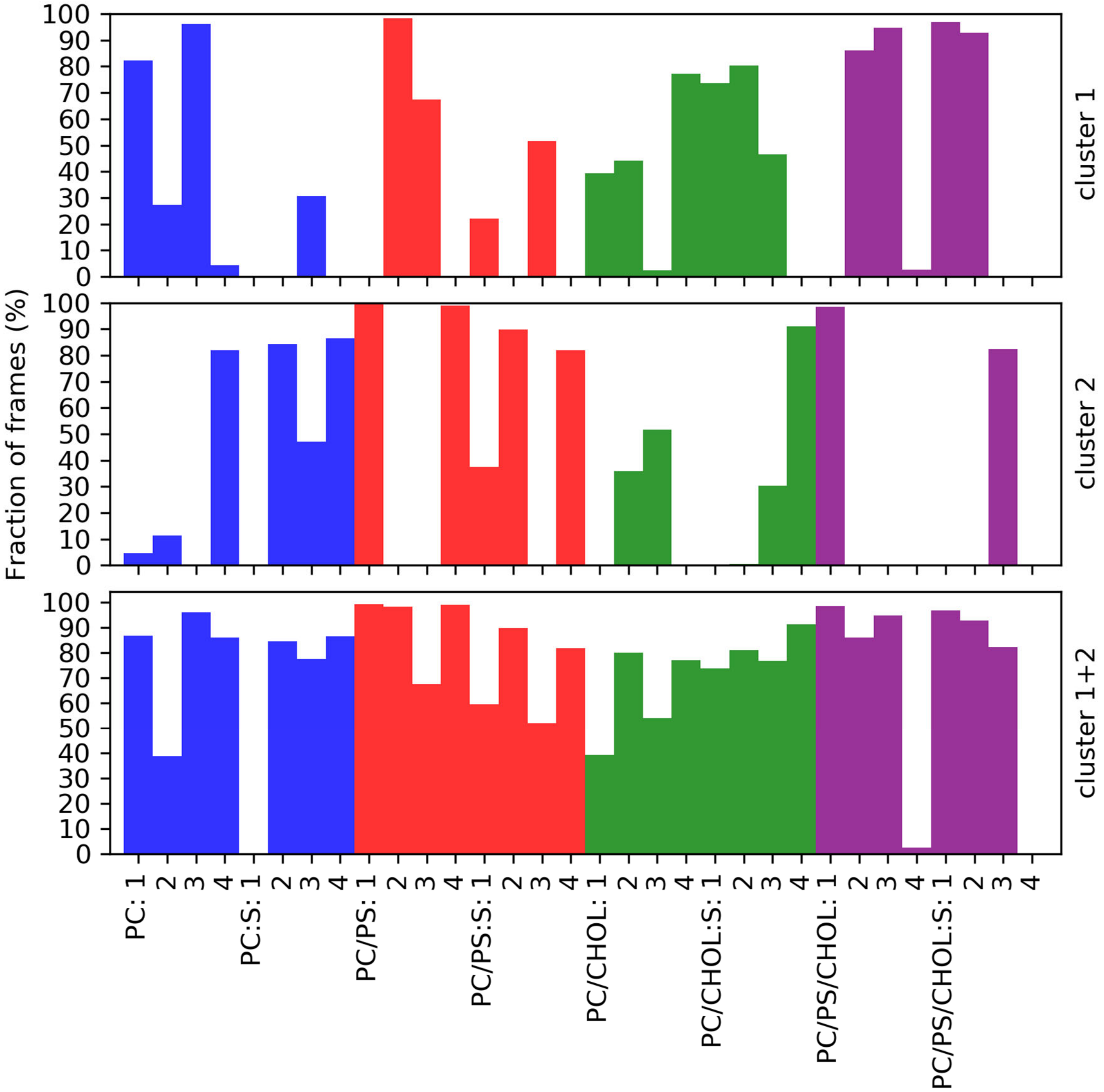

Supplementary Figure 3. Distribution of contributions to the two orientations of the N-terminal loop of IAPP from each simulation series.

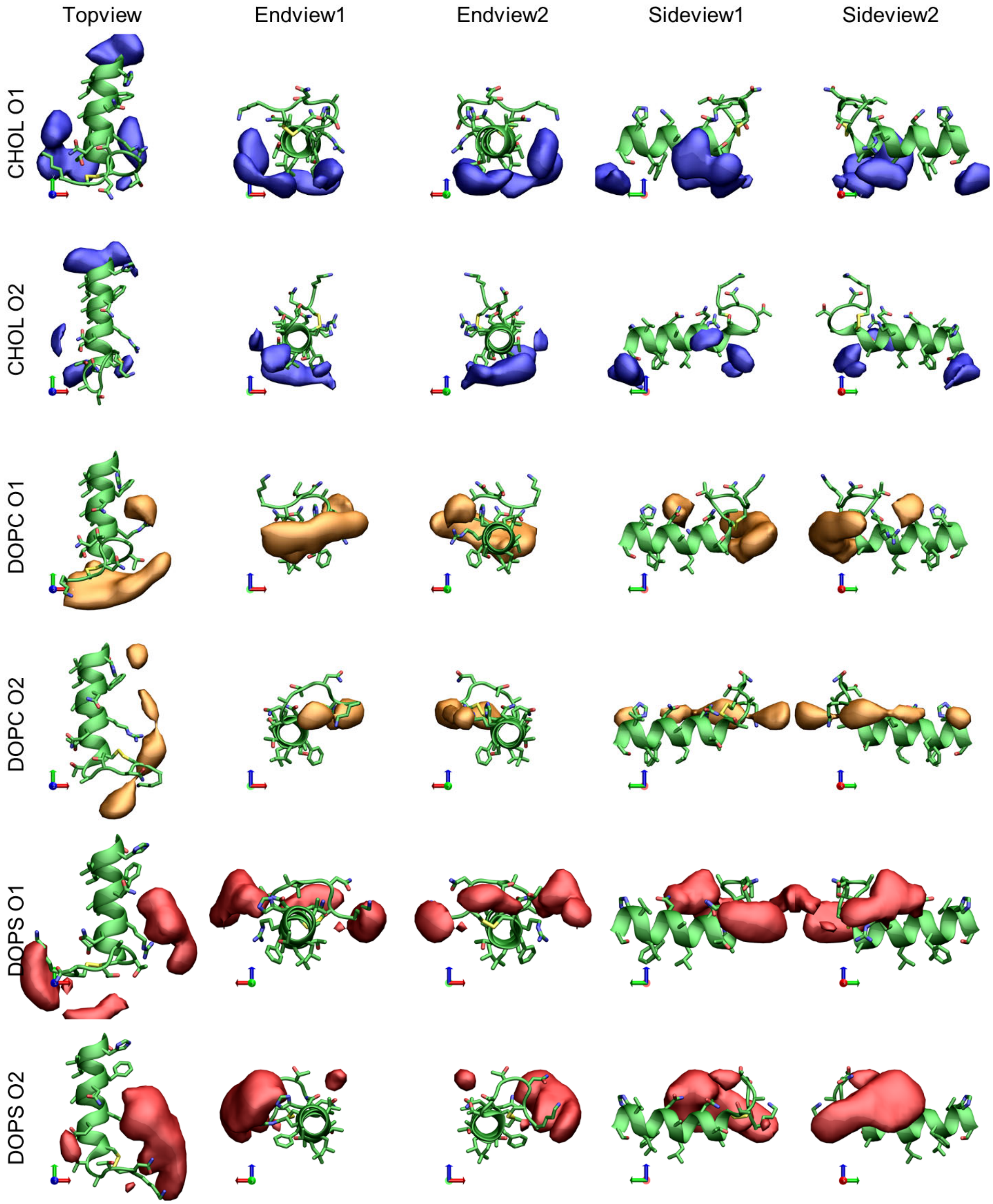

Supplementary Figure 4. 3D Occupancy maps of CHOL, DOPC, and DOPS around $IAPP_{1\text{-}19}$.

Distance based clustering of protein-lipid pairs

The protein-lipid pairs where clustered in a distance based manner, using the protocol illustrated in **Supplementary Figure 5**. All distances between selected lipid atoms and the protein atoms were calculated and transformed into a 1D array. An array was prepared for each simulation frame with a protein-lipid pair (with the lipid within 5Å of the protein). The arrays were clustered using K-means clustering to collect protein-lipid pairs with similar interaction patterns. The distances were measured using the GROMACS tool *gmx pairdist.* The data was processed using the *Numpy* package in *Python 2.7.* The k-means clustering was performed using the *scikit learn* module.

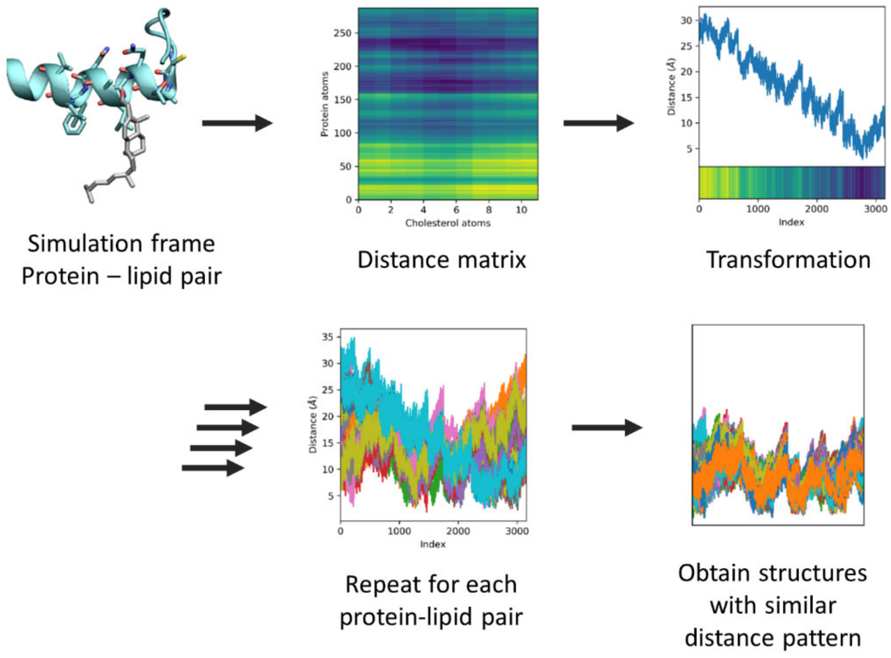

Supplementary Figure 5. 3D Occupancy maps of CHOL, DOPC, and DOPS around $IAPP_{1-19}$.

The number of clusters is decided based on the variance explained by the clusters, which was calculated from the centroids in the clustering. The clustering was performed separately for DOPC, DOPS, and CHOL; and separated for the two orientations of the N-terminal. For all series, the explained variance peaks between 8 and 11 clusters after which it levels off or stagnates, as shown in **Supplementary Figure 6**.

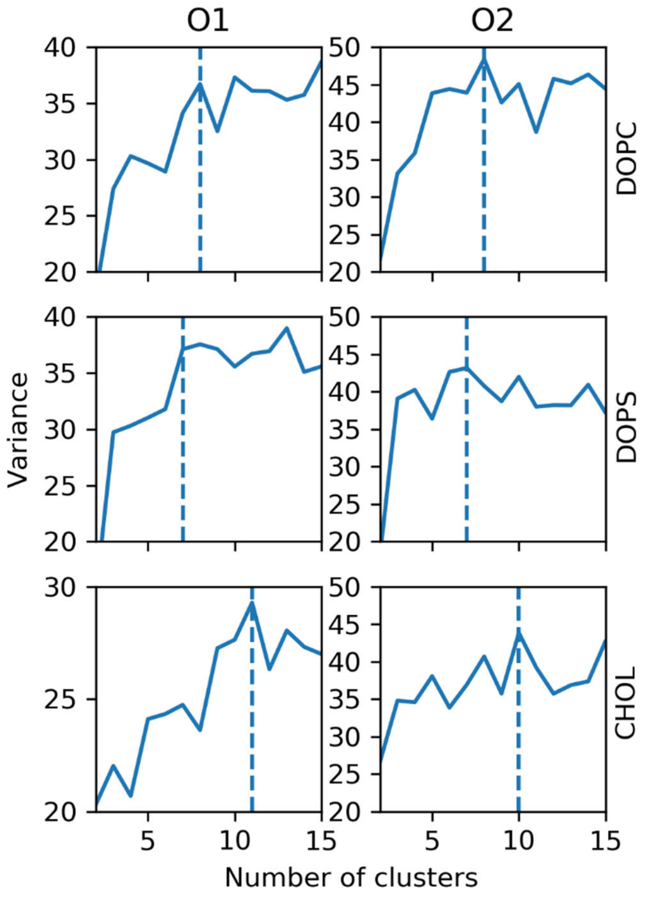

Supplementary Figure 6. Explained variance in the clusters.

The clusters were visually inspected and collected into general categories. One set of 10 categories of common binding modes was defined for the phospholipid and another for the cholesterol. The 5 largest categories of phospholipid and cholesterol binding modes are presented in the manuscript. The remaining are described here.

Phospholipids

	Binding mode	Residues	DOPC O1	DOPC O2	DOPS O1	DOPS O2
1	Between Arg11 and Phe15	R11, F15	15.9%	15.1%	13.2%	22.2%
2	Lys1	K1	9.0%	16.2%	17.8%	26.5%
3	Loop interaction	K1, C2, N3, T4	12.4%	18.9%	18.7%	8.3%
4	Between N-terminal and Arg11	K1, C2, C7, R11	0.0%	19.7%	0.0%	24.3%
5	Lys1 and left side helix	K1, A8, T9, Q10	12.0%	0.0%	22.8%	0.0%
6	Kink	V17, H18, S19	12.6%	8.3%	4.9%	8.3%
7	Between loop and Arg11	T6, R11	13.2%	0.0%	16.1%	0.0%
8	His18	H18	6.9%	12.2%	6.4%	0.0%
9	Between Gln10 and Asn14	Q10, N14	0.0%	9.4%	0.0%	9.3%
10	Helix end	C7, A8, T9	17.5%	0.0%	0.0%	0.0%

Supplementary Table 1: Explained variance in the clusters.

Binding mode 6:
This binding mode involves interactions with the kink-region, primarily His18 and Ser19.

Binding mode 7:
Binding between the loop and Arg11, this is the O1 counterpart of BM4.

Binding mode 8:
Interaction with His18.

Binding mode 9:
Interaction with Gln10 and Asn14. Only observed significantly in O2.

Binding mode 10:
At the helix end. Only observed for DOPC and in O1.

Cholesterol

	Binding mode	Residues	CHL1 O1	CHL1 O2
1	Kink region	F15, L16, V17, H18, S19	32%	26%
2	Thr9/Gln10	T9, Q10, L12	21%	12%
3	N-terminus	A8, T9, L12	10%	14%
4	Below helix	L12, F15, L16	8%	13%
5	Between loop and Arg11	A8, R11	9%	9%
6	Helix end	A6, C7, A8, T9	8%	0%
7	Loop interaction	N3, T4, A5, T6	0%	14%
8	Phe15	F15	0%	11%
9	Between Arg11 and Phe15	R11, F15	10%	0%
10	Lys1	K1	2%	0%

Supplementary Table 2: Overview of cholesterol binding modes, and the relative contributions from orientation.

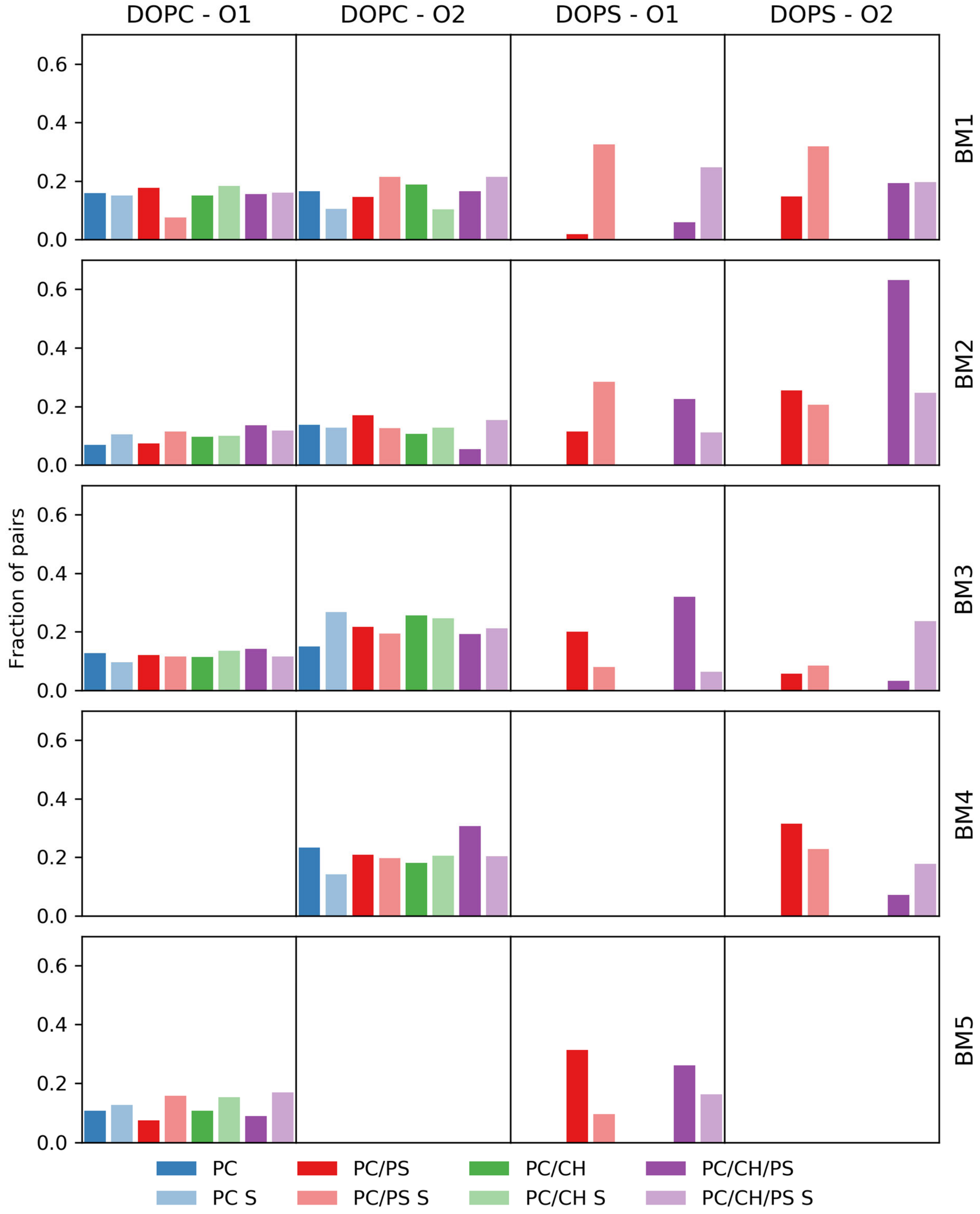

Supplementary Figure 7. Relative contributions from the individual simulations series to the each binding mode. For PC and PS lipids and each of the two N-terminal loop orientations.

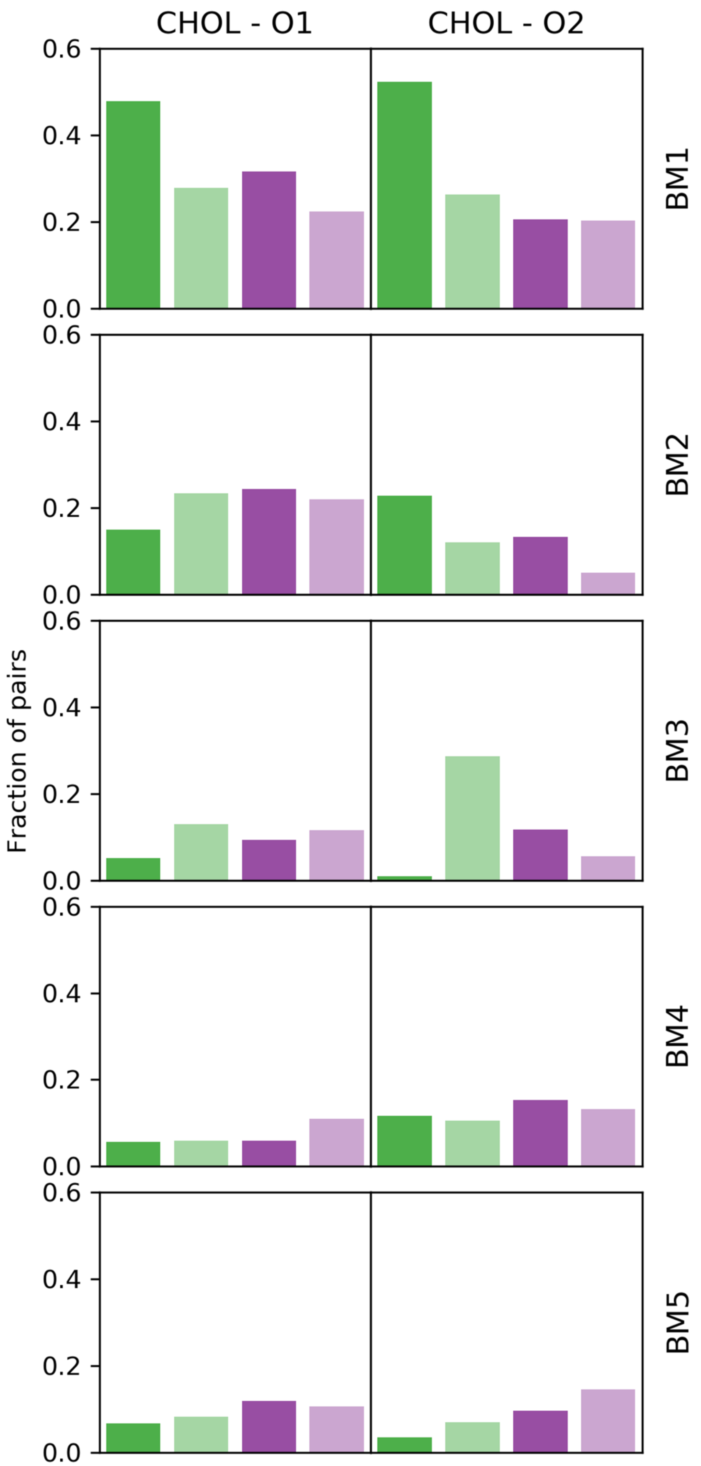

Supplementary Figure 8. Relative contributions from the individual simulations series to the each binding mode. For PC and PS lipids and each of the two N-terminal loop orientations.
